# Implementation and strategies to ensure adequate coordination within a Urology Department during the COVID-19 pandemic

**DOI:** 10.1590/S1677-5538.IBJU.2020.S122

**Published:** 2020-07-27

**Authors:** Francesco Esperto, Francesco Prata, Angelo Civitella, Karl H. Pang, Michele Marchioni, Piergiorgio Tuzzolo, Roberto M. Scarpa, Rocco Papalia

**Affiliations:** 1 Campus Bio-Medico University of Rome Department of Urology Rome Italy Department of Urology, Campus Bio-Medico University of Rome, Rome, Italy; 2 University of Sheffield Department of Oncology Sheffield United Kingdom, UK Department of Oncology University of Sheffield, Sheffield, United Kingdom, UK; 3 University of Sheffield Department of Metabolism Sheffield United Kingdom, UK Department of Metabolism, University of Sheffield, Sheffield, United Kingdom, UK; 4 University of Chieti Department of Medical Oral and Biotechnological Sciences “G D'Annunzio” Italy Department of Medical Oral and Biotechnological Sciences “G D'Annunzio” University of Chieti. Urology Unit SS Annunziata Hospital Chieti, Italy

**Keywords:** Clinical Protocols, COVID-19 diagnostic testing [Supplementary Concept], Telemedicine

## Abstract

**Purpose:**

to provide an update on the management of a Urology Department during the COVID-19 outbreak, suggesting strategies to optimize assistance to the patients, to implement telemedicine and triage protocols, to define pathways for hospital access, to reduce risk of contagious inside the hospital and to determine the role of residents during the pandemic.

**Materials and Methods:**

In May the 6^th^ 2020 we performed a review of the literature through online search engines (PubMed, Web of Science and Science Direct). We looked at recommendations provided by the EAU and ERUS regarding the management of urological patients during the COVID-19 pandemic. The main aspects of interest were: the definition of deferrable and non-deferrable procedures, Personal Protective Equipment (PPE) and hospital protocols for health care providers, triage, hospitalization and surgery, post-operative care training and residents' activity. A narrative summary of guidelines and current literature for each point of interest was performed.

**Conclusion:**

In the actual Covid-19 scenario, while the number of positive patients globally keep on rising, it is fundamental to embrace a new way to deliver healthcare and to overcome challenges of physical distancing and self-isolation. The use of appropriate PPE, definite pathways to access the hospital, the implementation of telemedicine protocols can represent effective strategies to carry on delivering healthcare.

## INTRODUCTION

A novel coronavirus was identified and considered responsible for a cluster of new cases of interstitial pneumonia in December 2019, in Wuhan, China. On February 11th, 2020, the disease caused by the SARS-CoV-2 virus (Severe Acute Respiratory Syndrome Coronavirus 2) was officially termed “COVID-19” by the World Health Organization (WHO) ( 1 ). The high potential of human to human transmission led to a rapid COVID-19 epidemic in China, and subsequently, the WHO declared COVID-19 as a global pandemic on March 11th ( 1 ). In Europe, Italy has been one of the most affected countries and the first one to adopt important restrictive measures on the whole national territory ( 2 ).

At the time of writing (May 7th, 2020), 3,833,547 total cases were reported. Of these, there were 2,261,992 symptomatic patients. Those in intensive care unit (ICU) represented 2%. Overall, 265,210 deaths have been reported in Italy. Meanwhile, the spread of the disease has dramatically increased in the USA, making it the leading country for total cases and total deaths ( 3 ).

All countries affected by COVID-19 are facing the major problem of ICU overcrowding and the progressive lack of resources. Many hospitals have to postpone major elective surgeries. Hospital departments worldwide limit procedures to urgent and non-deferrable cases, following the adoption of internal inpatients and outpatients management protocols. With the exponential increase in the number of cases, all countries had to reallocate medical resources to manage COVID-19 patients, with redistribution of medical and surgical activities ( 4 ).

To provide a snapshot of the current uro-oncological management in Europe during the COVID-19 emergency, Oderda et al. conducted a survey involving 57 European urological referral centers. They showed that the management of the main urological cancers has been altered dramatically by the COVID-19 pandemic, with most European centers (82%) declaring to be “much” or “very much” affected. Uro-oncological consultations for newly diagnosed cancers and follow-up were more than halved or almost suspended, in 55% and 71% of centers, respectively ( 5 ). Guidelines have been provided by major national and international scientific societies to aid physicians in the management of urological conditions during the COVID-19 outbreak.

We aim to summarize the current state of literature on the management of a Urology Department during the COVID-19 outbreak, suggesting strategies to optimize assistance to the patients, to implement telemedicine and triage protocols, to define pathways for hospital access, to reduce risk of contagious inside the hospital and to determine the role of residents during the pandemic.

## MATERIALS AND METHODS

On May 8th, 2020 we performed a review of the literature through online search engines (PubMed, Web of Science and Science Direct). We looked at recommendations on management of urological patients during the COVID-19 pandemic provided by the European Association of Urology (EAU) and the EAU Robotic Urology Section (ERUS). The main aspects of interest were: the definition of deferrable and non-deferrable procedures, Personal Protective Equipment (PPE) and hospital protocols for healthcare providers, triage, hospitalization and surgery, post-operative care, training and residents' activity.

A narrative summary of guidelines and current literature for each point of interest was performed.

### Deferrable and non-deferrable procedures

ICUs are being filled up rapidly, causing a shortage of hospital beds, mechanical ventilators and anesthesiologists. To decrease the general inflow of patients to hospitals, recommendations have been provided to reduce the number of medical and surgical procedures ensuring that only urgent and non-deferrable oncological surgeries are performed. On February 28th, the president of the Robert-Koch-Institute (RKI) suggested to defer all non-urgent surgeries ( 6 ). Similarly, in most of European National Health Systems a reduction of surgical activity was recommended. Several definitions of deferrable and non-deferrable procedures have been proposed. In particular, the EAU guidelines categorized procedures into priority groups ( Table-1 ):

1. emergency, life-threatening situations that cannot be postponed for more than 24 hours;
2. high priority, the last to postpone because of the concrete possibility of a clinical harm;
3. intermediate, should be cancelled but recommended not to postpone for more than 3 months. Clinical harm (progression, metastasis, loss of organ function) is possible if postponed 3-4 months but unlikely and;
4. low priority, that can be postponed for more than 6 months ( 7 ).

**Table 1:** Summary of EAU guidelines Office Rapid Reaction Group for oncological and non-oncological conditions ( 7 ).

	Priority	Condition	Treatment
**Oncological**	**Emergency**	Life threatening-organ function threatening condition	Cannot be postponed more than 24 hours.
	**High priority**	Clinical harm (progression, metastasis, loss of organ function and deaths) if postponed > 6 weeks	The last to cancel, prevent delay of > 6 weeks.
	**Intermediate priority**	Clinical harm possible (progression, metastasis, loss of organ function) if postponed 3 months but unlikely.	Not recommended to postpone more than 3 months.
			Reconsider in case of increase in capacity.
	**Low Priority**	Clinical harm very unlikely (progression, metastasis, loss of function) if postponed	Postpone up to 6 months
**Non-oncological**	**Emergency**	Life threatening situation	Cannot be postponed more than 24 hours.
	**High priority**	Clinical harm very likely if postponed > 6 weeks	The last to cancel, prevent delay of > 6 weeks.
	**Intermediate priority**	Clinical harm possible if postponed 3-4 months	Not recommended to postpone more than 4 months.
	**Low Priority**	Clinical harm very unlikely if postponed	Postpone 6 months

Stensland et al. ( 8 ) defined a list of urological conditions and surgical procedures that patients may undergo during the pandemic, stressing a more conservative approach whenever feasible. For example, benign prostate hyperplasia (BPH) and urinary tract stones should be treated only if complication occurs, with catheterization, and nephrostomy or ureteral stenting, respectively. Surgery should be maintained just for urological urgencies, such as testicular torsion, refractory gross hematuria and oncological disease (i.e. invasive muscle bladder cancer, suspected high grade T1 bladder cancer, kidney tumors >cT3) ( Table-2 ). The Research Urology Network (RUN) group has outlined priorities for urological patients ( Table-3 ), providing strategies for the management of urological patients not suspected of, or positive for COVID-19 ( 4 ). Treatments that ensure a fast discharge with the resolution of functional harms should be used. For instance, in cases of upper urinary tract obstruction, ureteral stents or percutaneous nephrostomy are preferred to more definitive procedures such as PCNL (Percutaneous nephrolithotomy) or RIRS (Retrograde intra-renal surgery). In cases of gross hematuria, surgery should be limited to cystoscopy for clot evacuation and concomitant hemostasis, preferably in an outpatient setting. However, bladder tumors should be removed if identified. The aim of these conservative approaches is to limit the need for blood transfusions and post-operative intensive care bed occupation. Considering the limited resources, urgent and emergent urological conditions are suggested to be treated under local or regional anesthesia whenever feasible to reduce aerosol generation ( 4 ).

**Table 2:** Summary of suggested triage of urological surgical cases during the COVID-19 pandemic by Stensland et al. ( 8 ).

	Condition	Pathology	Treatment Recommended	Comments
**Stensland et at** , ( 8 )	**Bladder cancer**	MIBC (regardless CHT)-refractory CIS (3rd line)	Radical cystectomy	5-8 days' hospital stay
		Suspected >cT1 BC	TURB	Outpatient procedure
	**Testicular cancer**	Suspected testicular cancer	Orchiectomy	Outpatient procedure
		Post-CHT LN (testicular cancer).	RPLN dissection - RT/CHT post-orchiectomy (if clinically appropriate)	Balance CHT (immunosuppression).
	**Renal tumor**	≥cT3 renal tumor	Radical nephrectomy + thrombectomy	
		cT1 renal tumor	Delay surgery / Ablative approach	
		cT2 renal tumor	Delay surgery up to 3 months	
	**Prostate cancer**	PCa high-risk	RT - Surgery (if ineligible for RT) -delay in selected cases	Most prostatectomy should be delayed
		PCa intermediate/low risk	Delay surgery	
	**Upper urinary tract cancer**	High grade ≥cT1 UTUC	Nephroureterectomy	1 - 4 days of hospital stay
	**Adrenal tumor**	Adrenal tumor >6 cm (suspected for carcinoma)	Adrenalectomy	0 - 1 day of hospital stay
		Adrenal tumor <6 cm.	Consider to delay	Possible rapid progression
	**Urethral/penile tumor**	Urethral/penile invasive or obstructive cancer	Limited data, consider partial penile penectomy, avoid LN dissection	Outpatient procedure
	**Endourology**	Stones	Nephrostomy/stent (preferable under local anaesthesia)	Emergency if obstructive/infected
		Indwelling ureteral stent	Delay most procedures (from 6-12 to 30 months)	Outpatient procedure
		BPH	Only if obstructive suprapubic/urethral catheter	
	**Female urology/ incontinence**	Urinary incontinence	Delay all procedures	High risk of infection
		Cystitis	Delay all procedures	
		OAB	Delay all procedures	
		Neurogenic Bladder	Delay all procedures	
		External nerve stimulator	Internalized or removed	
	**Reconstructive surgery**	Fistula with pelvic sepsis	Urine/fecal diversion (delay definitive repair)	
		Infected urinary sphincter	Explantation	
	**Urethral stricture**	Urethral obstruction	Suprapubic/urethral catheter	Outpatient procedure
	**Prosthetic surgery**	Penile prosthesis	Explant if infected	
	**General urology**	Priapism	Shunt	Outpatient procedure
		Spermatic cord torsion	Detorsion/orchidopexy	
		Refractary gross hematuria	Clot evacuation	
		Acute scrotal abscess and Fournier's gangrene	Surgery	
		Penile/testicular fracture	Surgery	
		Ureteral injury	Surgery	
		Bladder perforation	Surgery	
	**Transplant**	Renal transplant	Deceased donor, don't delay Live donor, delay	
	**Infertility**	Infertilty	Delay all procedures	

**MIBC** = muscle-invasive bladder cancer; **BC** = bladder cancer; **CHT:** = chemotherapy; **TURB** = trans-urethral resection of bladder; **LN** = lymphnodes: **RPLN** = retroperitoneal lymphnodes; **RT** = radiation therapy; **PCa** = prostate cancer; **BPH** = benign prostate hyperplasya; **OAB** = overactive bladder

**Table 3:** Summary of RUN group recommendations for urological conditions during Sars-CoV-2 era ( 4 ).

**RUN Group**	**Urgent**	Upper urinary tract obstruction/infection	Nephrostomy/stent (preferable under local anaesthesia)
		Acute urinary retention	Urethral/suprapubic catheter
		Clot retention	Cystoscopic clot evacuation - TURB/TURP
		Spermatic cord torsion	Manual derotation/surgery
		Infection of artificial sphincter/prothesis	Explant
		Scrotal abscess	Drainage
		Fournier's gangrene	Surgery
		Priapism	Corpora cavernosa aspiration/irrigation or Shunt (preferable under local anaesthesia)
	**Non-deferrable**	MIBC / refractory CIS	Radical cystectomy + Urinary diversion (high virus load in stool)
		NMIBC(>2cm/high grade)	TURB + intravesical therapy
		Testicular cancer	Radical orchiectomy
		Post-CHT retroperitoneal residual LN	Surgery
		cT3-T4 renal tumor	Radical nephrectomy ± thrombectomy
		cT2	Radical/partial nephrectomy
		High grade >cT1 upper urinary tract urothelial cancer	Nephrouretectomy + LN dissection
		High-risk/locally advance PCa unsuitable for RT or ADT	Radical prostatectomy + LN dissection
		>cT1G3 penile cancer	Partial penectomy ± groin LN dissection
	**Semi-non-deferrable**	PCa intermediate/high-risk	Radical prostatectomy
		NMIBC (<2cm/low grade)	TURB
		cT1b renal tumor	Radical nephrectomy
	**Deferrable**	cT1a renal tumor	Partial nephrectomy
		Uncomplicated urinary stones	Medical therapy
		BPH with LUTS	Medical therapy
		Urinary incontinence	Medical therapy
		Genitourinary prolapse	Medical therapy
		Male urethral disease	Medical therapy
		Prosthetic surgery	Medical therapy
		Infertility	Medical therapy
		Suspected PCa	Postpone prostate biopsy
		NMIBC follow-up	Postpone flexible cystoscopy
		Ureteral stent or Nephrostomy tube	Postpone replacement up to 6 months
		Low-grade NMIBC	Postpone intravesical therapy
	**Replaceable with other treatments**	High-risk/locally advanced PCa	RT or ADT (if cannot receive timely curative treatments)
		Small renal tumor	Ablative treatment not requiring general anaesthesia
		Testicular cancer + retroperitoneal LN	RT or CHT

**TURB** = trans-urethral resection of bladder; **TURP** = trans-urethral resection of prostate; **MIBC** = muscle-invasive bladder cancer; **NMIBC** = non-muscle-invasive bladder cancer; **CHT** = chemotherapy; **PCa** = prostate cancer; **BPH** = benign prostate hyperplasya; **LUTS** = lower urinary tract symptoms; **RT** = radiation therapy; **ADT** = andogen deprivation therapy; **LN** = lymphnodes

**Table 4:** Summary of COVID-19 task force actions regarding PPE for HWs ( 13 ).

Front Office staff working		Healthcare personnel in contact with patients					Laboratory staff in contact with biological samples
At station in direct contact with patients	At station with progressive glass	In contact with a suspected or confirmed case of COVID-19	In contact with a patient who presents symptoms of fever and / or cold and / or cough	Performing endoscopic procedures	Assigned to take a biological sample for COVID-19 + patient	Anesthesiologists performing intubation	
frequent hand hygiene by using 60 % alcohol solution	frequent hand hygiene by using 60 % alcohol solution	FFP2 filtering mask (use FFP3 only for the procedures that generate aerosols)	FFP2 filtering mask (use FFP3 only for the procedures that generate aerosols)	FFP3 filtering mask	FFP3 filtering mask	FFP3 filtering mask	FFP3 filtering mask
wear the FFP2 filtering mask during the entire work shift	/	goggles or visors to protect eyes from biological liquids' splashes	goggles or visors to protect eyes from biological liquids' splashes	goggles or visors to protect eyes from biological liquids' splashes	goggles or visors to protect eyes from biological liquids' splashes	goggles or visors to protect eyes from biological liquids' splashes	goggles or visors to protect eyes from biological liquids' splashes
wear protective glasses from liquids splashes during the entire work shift	/	water repellent PPE coat	/	water repellent PPE coat	water repellent PPE coat	water repellent PPE coat	water repellent PPE coat
provide a surgical mask, supplied at the desk, to be worn by the patient with visible respiratory symptoms	provide a surgical mask, supplied at the desk, to be worn by the patient with visible respiratory symptoms	double gloves	gloves	gloves	double gloves	double gloves	double gloves

The RUN group divided uro-oncological procedures into four categories: non-deferrable; semi-non-deferrable; deferrable; and replaceable with other treatments. Non-deferrable surgeries include muscle-invasive or high-risk progression bladder cancer, testicular cancer, renal tumor >T2, upper urinary tract cancer ≥cT1, high-risk prostate cancer unsuitable for radiation therapy (RT), and penile cancer >cT1G3 ( 4 ). For these pathologies, a delay could result in poorer cancer-related outcomes. If a hospital struggles with limited resources due to an uncontrolled COVID-19 spread, the patient should be transferred to a lower impact area for treatment. High-complexity surgery carries higher rates of morbidity and mortality and, in cases where patient's health is not jeopardized, it should be delayed ( 9 ). For selected patients not fit for major surgery, conservative approaches such as bladder-sparing treatments, may provide comparable oncological outcomes without affecting patients' comorbidities and safety ( 10 ). However, it has to be considered that the delay of surgical treatment of non-emergent oncological cases could lead to poorer standard oncological outcomes, affecting survival ( 11 ). In COVID-19 positive patients, non-emergent procedures should be postponed, while urgent surgeries have to be performed in a separated and dedicated operating theatre, following local institution recommendations for protection of the operating staff ( 11 ). Finally, all interventions for benign uncomplicated disease should be deferred until the end of the pandemic ( 4 ).

### PPE and hospital protocols for healthcare providers

The main goals for urologists and all health-care providers during the COVID-19 pandemic are to prevent patients from getting COVID-19, protect themselves as health care professionals, and deliver optimal urological care. To reach these goals, all medical personnel should comply with the PPE regulations. PPE includes: gloves, medical masks, goggles/face shield, gowns and aprons. For specific procedures, respirators (i.e. N95 or FFP2 standard or equivalent) are recommended ( 12 ). An adequate use of PPEs is essential to limit and contain the spread of the virus ( Table-4 ) ( 13 ). Effective preventive measures for the community, according to the WHO, include: performing hand hygiene frequently with a 60% alcohol-based solution avoiding touching eyes, nose, and mouth; practicing respiratory hygiene by coughing or sneezing on to the bent elbow or tissue; wearing a surgical mask and performing hand hygiene after its disposal; maintaining the social safe distance (a minimum of 1 meter) ( 12 ). To keep the risk of infection as low as possible, it is important to monitor temperature with thermoscan before each work shift, use PPE correctly and perform periodic swab for all health care providers ( 14 ).

### Triage

Hospitals should be divided into COVID-19 free and COVID-19 hospitals. The aim of triage is to stop any possible COVID-19 positive patient to access a COVID-19 free hospital. Accordingly, triage should be organized in hierarchic parts. Firstly, a telephone interview is required to enquire about clinical history, such as the presence of flu symptoms, sore throat, cough, fever, cold, intestinal symptoms and dyspnea within 3 weeks, and also about epidemiological history, such as a direct contact with a positive COVID-19 patient or origin from a red zone area. If there are no suggestions of a possible COVID-19 infection, the patient can be accepted to the hospital for the second phase of triage. At this stage, the patient is asked to wear a surgical mask, protective gloves and to follow all the recommended hygiene rules. The patient will then undergo thermoscan for the evaluation of the body temperature and all pre-hospitalization tests will be performed including chest x-ray and pharyngeal swab for COVID-19. Since most of the elective procedures are performed for malignant pathology it will be important, as far as staging is concerned, to strictly follow the guidelines thus avoiding non-essential tests, a valid aid to maintain the safety distance between patients. Simonato et al. proposed reducing the number of beds per room and/or to ensure the minimum safety distance between beds ( 15 ).

### Hospitalization and surgery

Hospital transmission was reported to be responsible for 41% of the nosocomial SARS infection ( 16 ). To prevent the spread of COVID-19 among healthcare providers, all staff members should be monitored with periodic swabs and, when serology tests become available, should undergo serology testing. For inpatients, social safe distance should be granted with all beds at least one meter away from each other. Since there is no vaccine nor cure for SARS-CoV-2, the spread of the virus should be stopped by preventing close contact ( 17 ). The spread from dry surfaces contaminated with secretions of infected people has been proven in previous studies ( 18 ). For this reason, an accurate cleaning of surfaces, following local hospital recommendations, has to be done systematically.

Elective surgeries have been cancelled to prevent any potential risk of infection of the patient and surgical team. Research protocols and experimental treatments have to be avoided and surgeries must be performed by skilled surgeons according to the standard approach in order to reduce operative time, post-operative complications and to spare resources. Any kind of surgery may increase the transmission risk of respiratory tract infections that could induce life-threatening outcomes, in case COVID-19 diagnosis is missed ( 19 ). For this reason, during intubation and extubating, the surgical team should wait outside the operating room, and all intubation maneuvers should be performed in negative-pressure operating theatre wearing appropriate PPE ( 20 ). Operative rooms usually have positive pressure technology in their aseptic zone (operating area) and are separated only by doors. These sliding barriers imply that the laminar air flow will be disrupted once doors are opened letting particles and aerosols to circulate freely. That is why it has been recommended to set up operating rooms at negative pressure to reduce COVID-19 dissemination beyond the theatre. The more people in the operating room, the more air-turbulences could worsen, regardless of the positive or negative pressure system ( 21 ). Therefore, there is the need to reduce the surgical team number to the minimum. Urologists were, and are pioneers of minimally invasive surgery (MIS): from endoscopy to robot-assisted laparoscopic surgery. MIS has been shown to reduce post-operative complications and peri-operative blood transfusions when compared to the open approach ( 22 ), supporting the need to limit the use of blood derivatives due to the decrease in blood donation. In order to spare resources, MIS should be performed where possible, by experienced surgeons outside of their learning curve ( 4 ).

Until now, there is little evidence on the differences in the risk of virus spread between MIS and open surgery ( 23 ). The possibility of theatre staff contamination during open, laparoscopic or robotic surgery is of a concern in case of a positive patient. Measures to reduce aerosolization in the operating room, such as insufflators continuous cycle, closed circuits fume extraction and performing surgeries at the lowest intraabdominal pressure allowed, should always be considered. Avoiding the use of two-way pneumoperitoneum insufflators is suggested to prevent the colonization of circulating aerosol in the insufflator or pneumoperitoneum circuit ( 24 ). Even if previous research has shown that laparoscopy promotes the aerosolization of viral pathogens present in the blood ( 25 – 27 ), currently, there are no specific data proving an aerosol spread of the SARS-CoV-2 during minimally invasive abdominal surgery ( 24 ).

It is known that any form of electrosurgery can produce smoke, with a potential of aerosolization. Li et al. showed that only 10 minutes using ultrasonic or electrical equipment during laparoscopy was sufficient to have a significantly higher particle concentration of the smoke compared to open surgery ( 28 ). Gas has a low mobility in the pneumoperitoneum, and this leads to an accumulation of aerosol formed during procedure in the abdominal cavity. A sudden release of trocar valves, larger skin incisions or incorrect trocar removal before the complete disinflation can expose the theatre staff to potentially infected pneumoperitoneum aerosol ( 23 ). Thus, operating room staff must confirm the complete and correct disinflation of the pneumoperitoneum at the end of every procedure. Otherwise, the proven benefits of MISs in terms of reduced post-operative complications and length of stay, as well as the advantages of ultrafiltration of most or all aerosol particles, must be strongly considered. Filtration of aerosolized particles can be more difficult during open surgery ( 26 , 27 ).

### Post-operative care

During the post-operative phase, the hospital stay should be reduced to the minimum without compromising patients' health. The aim is to discharge patients early, avoiding the onset of post-operative complications or even hospital readmission. In an ideal COVID-19 free hospital, patients should have undergone at least one nasopharyngeal swab with negative result before returning home. With regards to triage, post-operative care should be performed remotely whenever possible: lower infection rates among the staff and reducing patients contact are the main purposes to pursue ( 29 ). Laboratory values and pathological reports could easily be sent by e-mail, followed by a phone consultation and discussion. Cremades et al. found no difference in clinical results, and a similar number of patients required extra visits after the initial follow-up ( 30 ). Analogue results have also been shown in other previous studies ( 31 , 32 ).

### Training

The COVID-19 outbreak has led to cancelation or minimization of all elective major deferrable surgeries ( 33 ). In Italy and Spain, patients with scheduled oncological interventions were moved to hospitals considered COVID-19 free ( 13 , 33 ). Even face to face and diagnostic activities underwent a great reduction, and in some cases a complete cancellation. The COVID-19 pandemic will have a profound effect on surgical education for the foreseeable future. The Centers for Disease Control and prevention recently recommended avoiding any gatherings with more than 10 people ( 34 ). As a result, face to face academic activities, including teaching conferences and simulation labs should be avoided. The rotations between different institutions and abroad fellowships have been limited or cancelled, as rotating through different hospitals may significantly increase the risk of contagion for residents, patients, and other healthcare personnel. In addition, national and international urological conferences, such as the EAU and the American Urology Association (AUA) congresses have been postponed, cancelled or converted to a telematic format ( 35 ). The EAU guidelines, the American College of Surgeons (ACS), and even many government institutions, are suggesting to cancel elective surgery ( 7 , 36 ) and most facilities are minimizing participants in any operation to essential personnel only. A recent survey conducted by Amparore et al. showed an overall decrease in daily residents' exposure. Overall, 41.1% experienced a reduction of on call duties, 81.2% of ambulatory visits, 74.1% of diagnostic procedures, 62.1% of endoscopic surgery, 57.8% of open surgery and 44.2% of MIS. This decrease was even more pronounced for last year trainees ( 37 ).

In some countries, such us Italy, France and UK redeployment of urology residents has occurred allocating them to work on medical wards or ICU. Furthermore, the debate on the participation of trainees in clinical activity during the COVID-19 outbreak is still open. In some countries, tutors and educators suggest residents to stay home and step down if they are not required for any clinical or ward duties ( 38 ). Many residency programs have responded to the pandemic by assembling rotating teams to cover their urology services, reducing the risk of COVID-19 exposure to patients and residents alike ( 39 ). These factors will undoubtedly decrease resident case volume and will impact strongly on every aspects of their training. However, it is of note that health crisis could lead to an opportunity for trainees to improve skills not acquirable during the normal practice: how to manage urology patients during a pandemic.

In this scenario to avoid a complete slowdown of the residents' training and a possible burnout, that is already relatively high compared to other specialties ( 39 ), it is important to introduce new and alternative teaching methods such as smart learning. Webinars, podcasts, prerecorded sessions, social media and platforms, such as the EAU education section ( https://uroweb.org/education/online-education ) and the EAU Surgery in Motion School ( https://surgeryinmotion-school.org/ ) are all important tools to reduce the effects of the SARS-CoV-2 pandemic on residents training and to continue with the theoretical learning.

## CONCLUSIONS

In the current COVID-19 pandemic, while the number of positive patients globally are rising, it is fundamental to embrace a new way to deliver healthcare and to overcome challenges of physical distancing and self-isolation. In this review, we provided an insight into the COVID-19 overall situation and presented a picture of the current state of art in terms of the impact on urological patients, surgeons and trainees, providing practical recommendations.

Telemedicine is playing a crucial role because it can be used to support patients during an infectious pandemic to minimize contacts and the risk of SARS-CoV-2 exposure, reducing unnecessary hospital access, empowering patient's self-care, and also maintaining resident training. Even if the containment of the pandemic burst is currently the main purpose of all countries health and economic systems, we can't lose the focus on maintaining the best standard of care for non-urgent pathologies. A problem that we will soon have to cope with is the accumulation of cases delayed during this pandemic and the consequent extent of surgical waiting lists. A precise subdivision of hospitals into COVID-19 positive and COVID-19 free, and strictly following hygiene precautions will allow urological surgical activity to carry on, reducing the number of postponed cases.
